# Common features of neurodegenerative disease: exploring the brain-eye connection and beyond (part 2): the 2021 pre-symposium of the 15th international conference on Alzheimer’s and Parkinson’s diseases

**DOI:** 10.1186/s13024-022-00571-7

**Published:** 2022-11-01

**Authors:** Sharyn L. Rossi, Preeti Subramanian, Guojun Bu, Adriana Di Polo, Todd E. Golde, Diane E. Bovenkamp

**Affiliations:** 1 BrightFocus Foundation, 22512 Gateway Center Dr, Clarksburg, 20871 MD USA; 2 Department of Neuroscience, Mayo Clinic, Jacksonville, FL USA; 3 Departments of Neuroscience and Ophthalmology, Centre de recherche du Centre Hospitalier de l’Université de Montréal (CRCHUM), University of Montreal, Montreal, QC Canada; 4 Departments of Neuroscience and Neurology, Norman Fixel Institute for Neurological Diseases, Center for Translational Research in Neurodegenerative Disease, Evelyn F. and William L. McKnight Brain Institute, University of Florida, Gainesville, Fl USA; 5 Departments of Pharmacology & Chemical Biology, and Neurology, Center for Neurodegenerative Disease, Emory University, School of Medicine, Atlanta, GA USA

**Keywords:** Alzheimer’s disease (AD) and related dementias, Glaucoma, Age-related macular degeneration (AMD), Vascular cognitive impairment and dementia (VCID), Cerebral amyloid angiopathy (CAA), Neurodegeneration, Cellular senescence, Aging, Maintenance, Vascular contributions, Neurovascular unit, Blood brain barrier (BBB), Blood retina barrier (BRB), Thrombosis, Ischemic, Stroke, Vasodilation, Vasoconstriction, Retinal ganglion cells (RGC), Endothelial cells, Skeletal cells, Axon, Pericytes, Inter-pericyte tunneling nanotubes (IP-TNT), Astrocytes, Microglia, Hippocampal atrophy, Inflammation, Genetic, Intraocular pressure, Biomarker, Electrophysiology, Imaging

BrightFocus Foundation sponsored the third iteration of the “Common Features of Neurodegenerative Disease: Exploring the Brain-Eye Connection and Beyond” pre-conference symposium that preceded the 15th International Conference on Alzheimer’s and Parkinson’s Diseases (AD/PD 2021). Part 1 of the meeting report was previously published in Molecular Neurodegeneration. This completes the description of the meeting with Part 2.

**Session III, “Why are the vascular components and blood-brain/blood-retina barriers of the brain and eye important contributors to the tipping point between health and disease?”** was chaired by Dr. Adriana Di Polo.

Both the brain and the eye are highly vascularized organs that contain a dense capillary network. The neurovascular unit (NVU) is a community of cells including neurons, astrocytes, endothelial cells, pericytes, and microglia, that match blood supply for high metabolic demand and regulate the blood brain barrier (BBB) and blood retinal barrier (BRB) functions. The NVU ensures oxygen and nutrients are available to highly metabolic neurons and provides a method of waste removal.

Despite recent advances in our understanding of the pathology of cognitive and retinal degenerative diseases, the actual triggers that lead to neurodegeneration are currently unknown. One of the ‘tipping points’ for the onset and progression of pathology can be dysfunction of the microvasculature, dysregulation of neurovascular coupling, and alterations of the blood-brain/blood-retinal barriers. AD, glaucoma, and macular degeneration all feature vascular abnormalities, but the full extent to which these factors contribute to the development of disease is not well understood. The four talks in this session explored the molecular and cellular mechanisms that could be exploited to better understand the role of microvascular components in the pathophysiology, risk assessment, diagnostic, and novel therapeutic targets for brain and eye diseases.

An area of utmost importance when studying the NVU is an applicable, relevant model system. Mouse models serve an important purpose to investigate how genetic factors or cardiovascular risk factors may contribute to neuropathology, however, it is difficult to isolate and perturb specific elements of the NVU in living animals. Cheryl Wellington, PhD, University of British Columbia, developed a bioengineered human cell-based arteriole NVU [1]. This model system contains an anatomically correct configuration of endothelial cells, smooth muscle cells, astrocytes, and neurons scaffolded around a lumen, complete with dynamic perfusion achieved by a pulsatile reactor that drives circulation. This multi-layered system allows investigators to examine cross-talk between specific cell types that comprise the NVU and tease apart how alterations in different components may contribute to amyloid and tau deposition and their role in neurodegeneration. This dynamic system is ideal for drug development by providing a platform for screening therapeutic compounds while assessing efficacy and potential vascular side effects that may otherwise go undetected.

Glaucoma patients suffer from vascular deficits including decreased blood flow in the retina and optic nerve. To understand the mechanisms leading to these abnormalities, Luis Alarcon-Martinez, PhD, University of Montreal (Di Polo laboratory) (current address: Centre for Eye Research Australia), investigated whether pericytes play a role in microvascular dysregulation in optic neuropathies. He developed a minimally invasive 2-photon retinal live imaging technology to examine capillary hemodynamics at pericyte locations and quantify single-capillary blood flow. Using this technique, he and his colleagues identified nanotube-like processes that connect two pericytes on separate capillary systems forming a functional network in the mouse retina. These structures were named inter-pericyte tunneling nanotubes (IP-TNTs), a novel conduit for pericyte-to-pericyte communication that are crucial for neurovascular coupling in the retina [2]. Dr. Martinez showed that IP-TNTs are damaged during ischemic and glaucomatous insults through a mechanism that involves excessive calcium influx into pericytes. When pathological calcium influx to pericytes was restricted, IP-TNTs were preserved, and neuronal function restored leading to enhanced survival. This study identifies pericytes as promising therapeutic targets for glaucoma and potentially other optic neuropathies.

The role of pericytes, calcium signaling, and neurovascular coupling was further elucidated by the work of Anusha Mishra, PhD, Oregon Health and Science University, demonstrating that different signaling mechanisms control arteriole versus capillary cerebral blood flow and are differentially mediated by astrocytes and pericytes. Vasodilation of arterioles is achieved through a nitric oxide dependent neuronally-driven mechanism, while vasodilation of capillaries occurs through astrocytic AMPA-mediated calcium release and activation of purinergic receptors that release prostaglandins, dilating capillaries through pericytes [3]. Following ischemic stroke, the risk for developing dementia increases 4-fold and the rate of cognitive decline is significantly increased. Dr. Mishra’s research shows that astrocytes become hyperactive after stroke, releasing inflammatory molecules (e.g. 20-HETE) that cause vasoconstriction and override prostaglandin-induced dilation [4]. This is particularly important for disease prognosis because silent cerebral infarcts increase the risk of dementia and are common in people with AD. It has been shown that changes in the vasculature precede amyloid accumulation and may be used as a strategy for early detection of AD.

In addition to changes in individual vessels and the NVU, the BBB tightly regulates the composition of the neuronal environment necessary for normal synaptic function and thereby, information processing. Dysfunction of BBB proteins, transporters, receptors, and ion channels lead to breakdown and vascular leakage with subsequent neurodegeneration. Berislav Zlokovic, PhD, University of Southern California, has shown that BBB breakdown (BBBb) is accelerated in normal aging in the hippocampus and medial temporal lobes and is more evident in those who develop mild cognitive impairment (MCI).

Although amyloid and tau are both neurotoxic and vasculotoxic, BBBb and progression of cognitive impairment is independent of amyloid and tau status [5]. Multiple neurotoxic pathways can lead to neurodegeneration including the extravasation of red blood cells that increase reactive oxygen species, release of fibrinogen leading to activation of microglia, and other blood-associated factors like thrombin, plasminogen, albumin, and autoantibodies that disrupt the integrity and function of the BBB. APOE4 carriers display greater BBBb in the hippocampus and surrounding brain regions and this occurs prior to the first signs of cognitive impairment as well as hippocampal atrophy [6]. Because BBBb precedes other detectable pathological changes, biomarkers such as platelet-derived growth factor receptor beta (PDGFRb), can serve for early detection prior to the onset of cognitive impairment facilitating early therapeutic interventions.

The talk by Randy Kardon, MD, PhD, Carver College of Medicine, introduced the vasculature of the eye, highlighted the physiological and pathological similarities in the ocular and brain circulations, and discussed recent innovations that enable visualization of the capillaries without injectable dyes. One of these technologies is optical coherence tomography angiography (OCTA) that Dr. Kardon’s team has used to investigate peripapillary perfused capillary density in six chronic optic neuropathies including glaucoma, attributing decreased blood flow in glaucoma patients to the loss of neurons [7]. While OCTA has the limitation of not quantitatively detecting the dynamics of blood flow, Laser Speckle Flowgraphy (LSFG) is another relatively new non-invasive and quantifiable technology to study the dynamics of blood flow simultaneously in various regions of the eye. This technology allows studying the same eye for the presence and absence of metabolic activation and neuronal coupling. Dr. Kardon has successfully used this technology in pre-clinical models of ocular hypertension to measure blood flow and its dynamics to changes in perfusion pressure and metabolic demand [8].

**The live discussion opened with the question: what are the common features of vascular dysregulation in neurodegenerative diseases?** The success of having engineered models that incorporate pericytes to mimic neurovascular units has been challenging due to the size of the scaffolds and the need for a large number of cells. Each model system, though not perfect, has its disadvantages and advantages. Efforts are ongoing to preserve and study complex neovascular coupling in engineered models. The key to linking models and the human or animal brain is selecting the right models to the right questions and performing parallel studies mimicking human studies and animal models.

The panel discussed the role of IP-TNTs in brain and brain diseases. Although primarily reported in the retina, these structures have been reported in the developing and adult brains and have been shown to play a role in neurodegeneration [9]. Although there might not be an overlap in the role of pericytes between the retina and the brain, there might be parallel events. Several labs have shown that neuronal dysfunction precedes the pericyte loss caused by hypoxia and ischemic damage which then causes barrier dysfunction, a mechanism that seems to underline many of the degenerative conditions.

**Where to make an impact in vascular components of neurodegenerative disease?** The dogma in the field is that neuronal changes are first, followed by the vascular changes at the early stages of the disease. There is obligatory loss of vessels in different optic neuropathies whenever there is axonal loss. So, the question remains whether the changes in neurovascular coupling are causative or a result of the damage. The roles of risk and protective genes may be reversed. Studying this in the brain can be challenging but it is relatively simpler in the eye. Would restoring the blood flow be sufficient to restore neurovascular coupling? And what are the roles of genetic and environmental factors that can contribute to this? The possibility that the changes in the vascular contribution can be in the initial and later stages need further studies.

The session closed with discussing how the vasculature changes during healthy aging. Studies have shown that with age, the integrity of blood vessels is compromised, and more vascular leakage takes place. These changes are more pronounced in the brain regions affected by cognitive decline. This phenomenon may not be unique to the brain but may also affected in other organs including liver, heart, and lungs as part of the normal aging process.

**In conclusion**, aging is the most important risk factor for developing diseases such as AD, glaucoma, and AMD. Mechanisms of aging also cause comorbid changes in the cardiovascular, endocrine, immune, and skeletal systems that can contribute to the development and progression of neurodegenerative diseases. As we begin to develop more advanced disease-tailored treatments, it is also important to identify common mechanisms of neurodegeneration across diseases for a more comprehensive, integrated approach to treating age-related conditions. Similar collaboratory efforts have recently been undertaken resulting in guidelines to facilitate cross-disease research moving forward [10]. Understanding connections and consequences of a complicated health status can on the one hand, help researchers develop broad acting therapies, and on the other, assist healthcare providers as they tackle personalized care.

In Parts 1 and 2 of this meeting report, three cross-cutting mechanisms of neurodegeneration have been highlighted for progressive thinking about how these common features can be leveraged to simultaneously treat a host of comorbid conditions. We know that tackling aging through lifestyle interventions is a first step to promoting health span and longevity but, if we can identify the underlying mechanisms to aging gone awry, we can potentially halt the transition to disease across many aging bodily systems. Moreover, we know that in the face of extreme risk due to age, one third of the population is resilient to neuropathological changes in the brain, providing us with a resource to identify and harness these protective mechanisms to stave off disease.

Cross-disease collaborations and efforts can also shed light on important differences that may be relevant for treatment purposes. For instance, the *APOE* e4 allele is the most potent genetic risk factor for the development of sporadic AD; however, the e4 allele has been shown to be protective in glaucoma and AMD. A similar inverse effect has been noted for the *APOE* e2 allele which is protective in AD but a risk factor for glaucoma and AMD. Therefore, there may be different approaches to treatment related to the APOE pathway in the brain and the eye.

Another advantage to thinking outside of the ‘disease silo’ is that one can avoid duplication of efforts and enrich discoveries in ways to benefit more than one field of research. For example, if a patient registry will ensure volunteering of donations of brains and eyes from the same person, at the same time as collecting blood and other tissues and correlated clinical data, this could be an invaluable resource to uncover new discoveries. Brain researchers should consider looking at the eyes and, vice versa, eye researchers should look at the brain in their projects, with the understanding that cross-collaborations between experts in multiple fields will be required since protocols and interpretation of data may be different. In addition, funders from disparate disease groups can pool resources to support more expensive end-stage drug development and trials, while not losing focus on understanding the fundamentals of disease and serving the affected individuals.

The purpose of publishing this meeting summary is to serve as a catalyst for new hypotheses and collaborations between scientists, clinicians, administrators, officials, politicians, and other important stakeholders at non-profit, for-profit and government funding agencies. Accelerating our progression towards better understanding of disease mechanisms, preventions, and treatments will ultimately benefit affected families and society as a whole.

## Data Availability

Not applicable.

## Abbreviations

20-HETE: 20-Hydroxyeicosatetraenoic acid.
Aβ: amyloid-beta/beta-amyloid.
AD: Alzheimer’s disease.
AI: artificial intelligence.
ALS: amyotrophic lateral sclerosis.
AMD: age-related macular degeneration.
AMPA: α-amino-3-hydroxy-5-methyl-4-isoxazolepropionic acid.
ApoE: apolipoprotein E.
ATP: adenosine triphosphate.
BBB: blood brain barrier.
BBBb: blood brain barrier breakdown.
BRB: blood retina barrier.
CAA: cerebral amyloid angiopathy.
CSF: cerebrospinal fluid.
GWAS: Genome-wide Association Studies.
IOP: intraocular pressure.
iPSC: induced pluripotent stem cells.
IP-TNT: inter-pericyte tunneling nanotubes.
LSFG: Laser Speckle Flowgraphy.
Na: sodium.
NO: nitric oxide.
NVU: neurovascular unit.
OCTA: optical coherence tomography angiography.
PD: Parkinson’s disease.
PDGFRb: platelet-derived growth factor receptor beta.
PET: positron emission tomography.
PS1: presenilin 1.
RGC: retinal ganglion cells.
SASP: senescence associated secretory phenotype.
TDP-43: TAR DNA binding protein 43.
TREM: triggering receptor expressed on monocytes.
VCID: vascular contributions to cognitive impairment and dementia.
